# Selective regulation of aspartyl intramembrane protease activity by calnexin

**DOI:** 10.1007/s00018-024-05478-8

**Published:** 2024-10-26

**Authors:** Whendy Contreras, Jody Groenendyk, Marc Gentzel, Pascal Y. Schönberg, Frank Buchholz, Marek Michalak, Bernd Schröder, Torben Mentrup

**Affiliations:** 1 Institute of Physiological Chemistry, Medizinische Fakultät und Universitätsklinikum Carl Gustav Carus, Technische Universität Dresden, Dresden, Germany; 2 Department of Biochemistry, University of Alberta, Edmonton, AB T6G 2H7 Canada; 3 Core Facility Molecular Analysis - Mass Spectrometry, Mass Spectrometry & Proteomics, Center for Molecular and Cellular Bioengineering (CMCB), Technische Universität Dresden, Dresden, Germany; 4 Medical Faculty, University Hospital Carl Gustav Carus, UCC Section Medical Systems Biology, TU Dresden, 01307 Dresden, Germany

**Keywords:** Intramembrane proteolysis, Calnexin, SPPL2c, Protease regulation, Male reproduction, ER quality control, Signal peptide peptidase

## Abstract

Signal peptide peptidase-like 2c (SPPL2c) is a testis-specific aspartyl intramembrane protease that contributes to male gamete function both by catalytic and non-proteolytic mechanisms. Here, we provide an unbiased characterisation of the in vivo interactome of SPPL2c identifying the ER chaperone calnexin as novel binding partner of this enzyme. Recruitment of calnexin specifically required the N-glycosylation within the N-terminal protease-associated domain of SPPL2c. Importantly, mutation of the single glycosylation site of SPPL2c or loss of calnexin expression completely prevented SPPL2c-mediated intramembrane proteolysis of all tested substrates. By contrast and despite rather promiscuous binding of calnexin to other SPP/SPPL proteases, expression of the chaperone was exclusively required for SPPL2c-mediated proteolysis. Despite some impact on the stability of SPPL2c most presumably due to assistance in folding of the luminal domain of the protease, calnexin appeared to be recruited rather constitutively to the protease thereby boosting its catalytic activity. In summary, we describe a novel, highly specific mode of intramembrane protease regulation, highlighting the need to systematically approach control mechanisms governing the proteolytic activity of other members of the aspartyl intramembrane protease family.

**Supplementary Information:**

The online version contains supplementary material available at 10.1007/s00018-024-05478-8.

## Introduction

The family of aspartyl intramembrane proteases (I-CLIPs) consists of the two presenilins that constitute the catalytically active core of the γ-Secretase complex as well as the five Signal peptide peptidase (SPP)/SPP-like (SPPL) proteases SPP, SPPL2a, SPPL2b, SPPL2c and the more distantly related SPPL3 protease [1]. While γ-Secretase is active as a heterotetrameric complex exclusively processing type I-oriented transmembrane segments, SPP/SPPL proteases typically require a type II topology of their substrates. Intriguingly, most of the five SPP/SPPL enzymes localise to different subcellular compartments, suggesting largely non-redundant functions. SPPL3 resides within membranes of the mid Golgi apparatus [2], while SPPL2a and SPPL2b can be found later in the secretory pathway, either in the endo-lysosomal system or at the plasma membrane, respectively [3, 4]. The only organelle that harbours two different SPP/SPPL proteases is the endoplasmic reticulum (ER), which represents the predominant localisation of SPP and SPPL2c [4, 5].

Over the last almost 25 years of SPP/SPPL-related research, our knowledge about the functions of these enzymes has significantly expanded. SPP is not only involved in the eponymous breakdown of signal peptides within the ER [6], but based on the cleavage of multiple identified substrates is also embedded in pathways impacting carcinogenesis [7, 8], ER stress responses [9] and metabolic regulation [10]. Furthermore, it controls the propagation of several viruses including the Hepatitis C virus [11]. SPPL3 modulates the cellular glycosylation status by controlling the abundance of various Golgi-resident glycosyltransferases [12]. SPPL2a, which is strongly expressed in the immune system, is essential for development and function of B and dendritic cells due to proteolysis of the cytotoxic CD74 N-terminal fragment (NTF) [13, 14]. Together with SPPL2b, it is also involved in the regulation of anti-fungal immune responses as well as in the prevention of atherosclerosis [15, 16]. Finally, SPPL2c, that by contrast to the other rather ubiquitously expressed family members, is exclusively and even transiently expressed during male gametogenesis, and impacts several aspects of sperm function [5, 17].

Typically, SPP/SPPL proteases are involved into two-step proteolytic processes called regulated intramembrane proteolysis (RIP). In this process, a single span type II substrate is first processed by an ectodomain removing protease. This generates a membrane-bound stub (NTF), that represents the actual substrate of intramembrane proteolysis and is then further cleaved by the responsible intramembrane protease [18]. In this model, regulation occurs at the first proteolytic event so that for a long time SPP/SPPL proteases were believed to function as slow, but constitutively active enzymes that do not require mechanisms to control their activity. However, this view was challenged by the notion that tail-anchored (TA) proteins, that have small luminal domains and therefore represent naturally short substrates, can be proteolysed directly by all family members except SPPL3 [5, 17, 19, 20]. In line with these findings, we have recently identified the small testis-specific Frey1 protein as a regulator of SPPL2c activity [21]. Frey1 specifically binds to the catalytic centre of SPPL2c using an inhibitory motif that can also be utilised to generate substrate-based inhibitors of other aspartyl I-CLIPs [22]. In cell-based overexpression systems, this leads to a complete block of SPPL2c activity against all tested substrates [21]. By contrast to this clear effect, the male infertility phenotype of Frey1-deficient mice [21, 23, 24] is not evoked by changes in the proteolytic activity of SPPL2c, but rather relates to function of this protein in the assembly of protein complexes required for sperm oocyte fusion. This function of Frey1 is supported by SPPL2c, that acts as a scaffold in this process and protects the intrinsically unstable Frey1 molecule from degradation by a yet unknown mechanism [21].

These findings prove that the activity of SPP/SPPL proteases can be modulated in a highly specific manner. Still, only very little is known about mechanisms controlling SPP/SPPL-mediated intramembrane proteolysis. Based on an unbiased analysis of the in vivo interactome of SPPL2c, here we characterise the ER chaperone calnexin, a crucial factor of the ER protein quality control, as novel interaction partner of SPPL2c. We demonstrate that binding of calnexin requires the N-terminal N-glycosylation of SPPL2c. In contrast to the SPPL2c/Frey1 interaction, presence of calnexin is essential for the intramembrane protease to acquire its activity. Despite rather unspecific recruitment of calnexin to all SPPL2 proteases and SPP, the ER chaperone is exclusively required for SPPL2c-mediated intramembrane proteolysis, highlighting the existence of specific pathways controlling the activity of individual SPPL proteases.

## Materials and methods

### Mice

Breeding of and experiments with laboratory mice were approved after ethical evaluation by the Landesdirektion Sachsen (TV A 12/2018, DD24.1-5131/450/12; TV vG 5/2023, 25-5131/564/5). SPPL2c-, Frey1- or double deficient mice on a C57BL/5 N background [5, 21] as well as *calnexin*^−/−^ [25] mice have been described before. Animal experiments including calnexin-deficient mice were carried out according to the University of Alberta Animal Policy and Welfare Committee and the Canadian Council on Animal Care Guidelines. The approval for use of animals in research was granted by the Animal Care and Use Committee for Health Science, a University of Alberta ethics review committee. The protocols were approved by the Committee (AUP297 and 409).

### Cell culture

HEK293T (DSMZ- German Collection of Microorganisms and Cell Cultures GmbH No.: ACC 635) and HeLa (DSMZ No.: ACC 57) were cultured in Dulbecco’s modified Eagle´s medium (DMEM, Gibco) supplemented with 10% fetal calf serum (FCS) and a mixture of penicillin (100 U/ml)/ streptomycin (100 µg/ml) and kept in humidified incubators with an atmosphere of 95% air and 5% CO_2_. For subcultivation, confluent monolayers were washed with phosphate-buffered saline (PBS) and then incubated with 1 ml Accutase for 5 min to detach cells. HEK293T cells were finally seeded in 24-well (for CRISPR/Cas9 editing), six-well (normal lysis), or 10 cm plates (immunoprecipitation assays) prior to transient transfection. HeLa cells were grown on sterile coverslips in 12 well-plates for immunofluorescence. Cells were negative for mycoplasma infection and tested on a regular basis by PCR.

### Generation of calnexin-deficient HEK293T cells

Calnexin-deficient lines were generated employing CRISPR/Cas9 technology. Three guide RNAs (sgRNA) targeting exons 5 and 7 were designed using the CC-top tool (Supplementary data, Table S2) and obtained from Synthego. Cas9 encoding mRNA was purchased from TriLink BioTechnologies. For transfection, 1 µl Lipofectamine MessengerMax (Invitrogen) was added to 25 µl Opti-MEM (Gibco), mixed well and incubated for 10 min at RT. In the meantime, 1 pmol Cas9 mRNA was added together with 10 pmol of each sgRNA to 25 µl of fresh Opti-MEM and combined with the lipofectamine mix. After 5 min, the resulting solution was added to 5 × 10^4^ HEK293T cells seeded into 24-well-plates the day before. Transfected cells were incubated for 72 h. Successful targeting of the CANX locus was validated by genomic DNA extraction using the QIAamp ^®^ DNA Mini and Blood kit. The obtained genomic DNA was used as a template for amplification of the targeted regions in exons 5 and 7 employing specific primers (Supplementary Table). Upon agarose gel extraction, the DNA fragment was purified using the High Pure PCR Product Purification kit (Roche) and prepared for sequencing carried out with the help of Microsynth Seqlab (Göttingen, Germany) employing the primers described in the Supplementary Table. Subsequently, single calnexin-deficient clones were isolated from the parental cell line by limited dilution. Calnexin-deficient clones were identified by Western blotting for calnexin.

### Plasmids and cloning

The constructs encoding wild type murine SPPL2c-myc and SPP-myc, its inactive mutant SPPL2c D457A and the glycosylation-deficient SPPL2c N106A-myc mutant as well HA-tagged SPPL2c substrates have been described previously in [5]. Plasmids encoding SPPL2a-myc, SPPL2b-myc and HA-VAMP2 have been described in [3] and [19], respectively. The SPP N10/20A-myc variant was generated from wild type SPP-myc by site directed mutagenesis. C-terminally 3xFLAG-tagged SPP, SPPL2a, SPPL2b and SPPL2c expression constructs were generated by PCR based on the respective myc-tagged plasmids. Plasmids encoding N-terminally HA-tagged versions of murine RAMP4-2, HO-1 and syntaxin-8 were described in [5] or [17], respectively. Orfs encoding murine calnexin and calmegin were amplified from murine testis cDNA and equipped with a C-terminal 3xFLAG tag. N-terminal chimeras between SPP and SPPL2c were obtained by overlap extension PCRs from the respective myc-tagged wild type open reading frames (orfs). Modified orfs were amplified with the Phusion DNA polymerase (Thermofisher Scientific) and ligated into the target vectors (pcDNA 3.1 hygro(+) or pcDNA4/TO, respectively prior to transformation of electrocompetent *Escherichia coli* XL1Blue cells (Stratagene). The nucleotide sequences of select clones was confirmed by sequencing (Microsynth Seqlab, Göttingen, Germany). Transfection-quality plasmid DNA was isolated using the PureYieldTM Plasmid Midiprep System (Promega).

### Transient transfection

24 h after seeding, cells were transfected with the indicated plasmids using polyethylenimine (PEI Max Transfection Grade Linear Polyethylenimine) in a DNA: PEI ratio of 1:2.5. When cells were transfected with several plasmids simultaneously, the DNA amount was evenly divided between the different plasmids. 6 h after addition of the transfection mixture to the cells, the culture medium was replaced. Cells were finally subjected to further analysis 24 h after transfection.

### Protein extraction and quantification

Cell monolayers were washed with PBS and cells detached with a cell scraper in PBS supplemented with the cOmplete (Roche) protein inhibitor mix. Subsequently, cells were pelleted by centrifugation at 1000xg for 5 min at 4 °C and immediately resuspended in lysis buffer (150 mM NaCl, 50 mM Tris-HCl pH 7.4, 1% Triton X-100, 0.1% SDS) in the presence of protease inhibitors (cOmplete, 0.5 µg/ml pepstatin A (Sigma-Aldrich), 4 mM Pefabloc SC Protease Inhibitor (Roth) 4 mM EDTA). For tissue lysis, testes were decapsulated and homogenised in lysis buffer without detergents. Upon homogenisation, Triton-X100 as well as SDS were added to the tissue homogenates to the final concentrations described above and mixed well. In all cases, lysates were subsequently sonicated and kept on ice for one hour before pelleting of remaining cellular debris for 10 min at 18000 xg. Finally, protein concentrations of the cleared supernatants was determined employing the Pierce BCA Protein Assay Kit (Thermo Fisher) according to the manufacturer’s recommendations.

### SDS-PAGE and western blotting

Protein lysates were prepared for SDS-PAGE using 5x SDS sample buffer (625 mM Tris-HCl, pH 6.8, 5% [w/v] SDS, 50% [v/v] glycerol, 500 mM dithiothreitol, trace amounts of bromophenol blue) and heated 5 min at 56 °C. Equal amounts of protein were separated at 100 V by standard SDS-PAGE employing a Tris-glycine buffer system using 10 and 12.5% polyacrylamide gels depending on the size of the proteins of interest. After electrophoresis, proteins were transferred to a Amersham Protran nitrocellulose membrane (VWR) by semidry transfer employing 65 mA/blot for 2 h. Nitrocellulose membranes were shaken for 1 h in blocking solution (5% non-fat milk (Roth) in TBS-T (137 mM NaCl, 2,7 mM KCl, 0,1% [v/v] Tween 20, 25 mM Tris/HCl pH 7.4)) followed by overnight incubation at 4 °C with the primary antibody diluted in blocking solution. Afterwards, membranes were washed three times for 10 min with TBS-T prior to incubation with the required peroxidase-conjugate secondary antibody (Dianova) also diluted in blocking solution. After three final washes with TBS-T, the proteins of interest were detected using the Amersham ECL Advanced Western Blotting Detection Reagent (GE Healthcare) and the Amersham Imager 600 RGB. The following primary antibodies were used for this project: anti-FLAG M2 (Sigma-Aldrich), anti-SPPL2c [5], anti-SPPL2a [3], anti-SPPL2b [26], anti-HA 3F10 (Roche), anti-myc 9B11 (Cell Signaling Technology), anti-cofilin D3F9 (Cell Signaling Technology), anti-GAPDH (W17079A, Biolegend), anti-calnexin (66903-1-Ig, proteintech), anti-Syntaxin-8 (R&D, AF5448) and anti-actin (abcam). An antiserum against the C-terminus of murine SPP (AETESKEESTEASASKRLEK) was generated by the Pineda Antikörper-Service (Germany).

### Generation of anti-SPPL2c/SPP sepharose beads

Anti-SPPL2c and anti-SPP beads were generated based on CNBr-activated Sepharose 4B (GE Healthcare) according to the manufacturer’s recommendations. Prior to the coupling procedure, SPPL2c and SPP targeting antibodies were concentrated using Vivaspin 20 centrifugal concentrators (Sartorius) with a molecular weight cutoff of 50,000 Da. The concentrated antibody solution was diluted in a final volume of 3,5 ml of coupling buffer (0.1 M NaHCO_3_, 0.5 M NaCl, pH 8.3) and coupled to 1 g of CNBr-activated Sepharose 4B for 2 h at room temperature (RT) under constant rotation. After a subsequent wash with coupling buffer, antibody-coupled beads were incubated with quenching buffer (0.1 M Tris-HCl, pH 8.0) for 2 h at RT, followed by three washing cycles with either 0.1 M acetic acid and 0.5 M NaCl (pH 4.0) or 0.1 M Tris-HCl, 0.5 M NaCl (pH 8.0). Finally, the beads were resuspended in 50 mM Tris-HCl (pH 7.4) until further use.

### Immunoprecipitation

The interaction between SPP/SPPL proteases and calnexin was analysed by immunoprecipitation (IP) assays. For this purpose, cells or tissues were lysed as described before but in the presence of 0.5% DDM (n-Dodecyl-B-D-Maltoside, GLYCON Biochemicals GmbH) instead of Triton-X100 and SDS. Protein concentrations were quantified by BCA and equal protein amounts were employed for immunoprecipitation. For this purpose, 500 µl of adjusted lysates were incubated with either 15 (anti-FLAG M2 beads, Sigma) or 25 µl (anti-SPPL2c/SPP beads) antibody-coupled beads overnight at 4 °C under constant rotation. Afterwards, beads were pelleted by centrifugation at 1000 xg for 1 min and washed five times with DDM-containing IP buffer. The purified immunocomplexes were eluted in 2x SDS sample buffer at 56 °C for 10 min. Samples were finally analysed by Western Blotting.

### Mass spectrometric identification of SPPL2c interaction partners

Testes from Wt and SPPL2c deficient mice were lysed in presence of 0.5% DDM DDM buffer, and one milligram of protein lysates was subjected to immunoprecipitation employing anti-SPPL2c sepharose beads. After the final washing step, beads were resuspended in 100 µl of 20mM Tris-HCl pH 7.4. Samples were digested on beads twice with 200 ng of trypsin overnight (Trypsin Gold, Promega, USA/ Germany) and subsequently with 100 ng of rLys-C (Promega, USA/ Germany). Protein digests were desalted with Ultra-Micro C18 SpinColumns (Harvard Apparatus, USA). Co-immunoprecipitated eluates were followed by mass spectrometry (IP-MS). Last, dried digests were recovered with 3 ml of 30% formic acid in water supplemented with standard peptide mixture (25 fmol/µl; Retention Time Calibration Mixture, Thermo Pierce, Germany) and diluted to a final volume of 23 ml with water; a total of 5 µl of this solution was injected for nano liquid chromatography-tandem MS (nanoLC-MS/MS). NanoLC-MS/MS analyses were performed with a Q Exactive HF mass spectrometer (Thermo Fisher Scientific, USA/Germany) hyphenated to Nanoflow LC system (Dionex 3000 RSLC, Thermo Dionex, Germany). Peptides were separated in a linear gradient of 0.1% aqueous formic acid (eluent A), and 0.1% formic acid in 60% acetonitrile (eluent B) in 120 min, and the mass spectrometer was operated in data-dependent acquisition mode (TopN 10). Raw files were imported into Progenesis QIP v4.2 (Nonlinear Dynamics, UK) software for analysis and quantification. Peptide and protein identifications were performed with Mascot v2.6 (Matrix Science, UK). Fold changes were calculated in Microsoft Excel. Detailed data about instrument settings are provided in Supplementary Material.

### Indirect immunofluorescence

Transiently transfected HeLa cells were washed three times with PBS and fixed with 4% w/v paraformaldehyde (PFA) in PBS for 20 min. Afterwards, cell permeabilisation was carried out by incubation in PBS-saponine (PBS + 0.2% saponine) for 5 min followed by quenching of remaining PFA for 10 min in quenching buffer (0.12% w/v glycine in PBS-saponine). Coverslips were kept for 1 h at RT in a blocking buffer (10% FCS v/v in PBS saponine) for blocking. Immuno-staining was performed by overnight incubation at 4 °C with primary antibodies diluted in blocking buffer. After five washing steps with PBS-saponine, coverslips were incubated for 1 h in blocking buffer supplemented with AlexaFluor-coupled secondary antibodies (Dianova). Finally, samples were washed five times with PBS-saponine and twice with ddH_2_O before mounting on microscopy slides using Mowiol 4–88 (Merck) containing 20 mg/ml 1,4-diazabicyclo (2,2,2)octane (DABCO, Sigma) and 1 µg/ml DAPI (4′,6-diamidino-2-phenylindole, Sigma) for visualization of nuclei. Pictures were acquired using a Leica SP5 or Leica STELLARIS confocal laser-scanning microscope and processed with ImageJ software (version 1.52a). For the quantification of HO-1 translocation, mean fluorescence intensity of a nuclear (nuc) and extranuclear (ex) region of interest were measured employing ImageJ and nuc/ex ratios were calculated using Excel 2016.

### Data analysis

Western blot images were analysed by densitometry using Image J (version 1.52a). Band densities were normalised to those of the protein used as a loading control, for example, cofilin or GAPDH. All data were normalised to the control sample to determine the substrate cleaving percentage. When wild type and calnexin-deficient HEK293T cell lines were analysed for the proteolytic activity of different SPP/SPPL proteases, the data were normalised to the substrate/loading control ratios of EV transfected samples for each line individually to minimise the effects of different transfection efficiencies on data interpretation. Calculations were performed using Excel 2016. By contrast, graphs and statistical analysis were carried out using the GraphPad Prism software version 8.4.3. The diagrams show mean values ± SD, and the statistical test employed is indicated in each Figure legend. N indicates the number of independent experiments while n stands for the number of independent samples. Figures and schemes were assembled using Adobe Illustrator CC software (version 2015.3.1). Western Blot as well as immunofluorescence images were edited using GNU Image Manipulation Program software (GIMP, version 2.10.14).

## Results

**Fig. 1:**
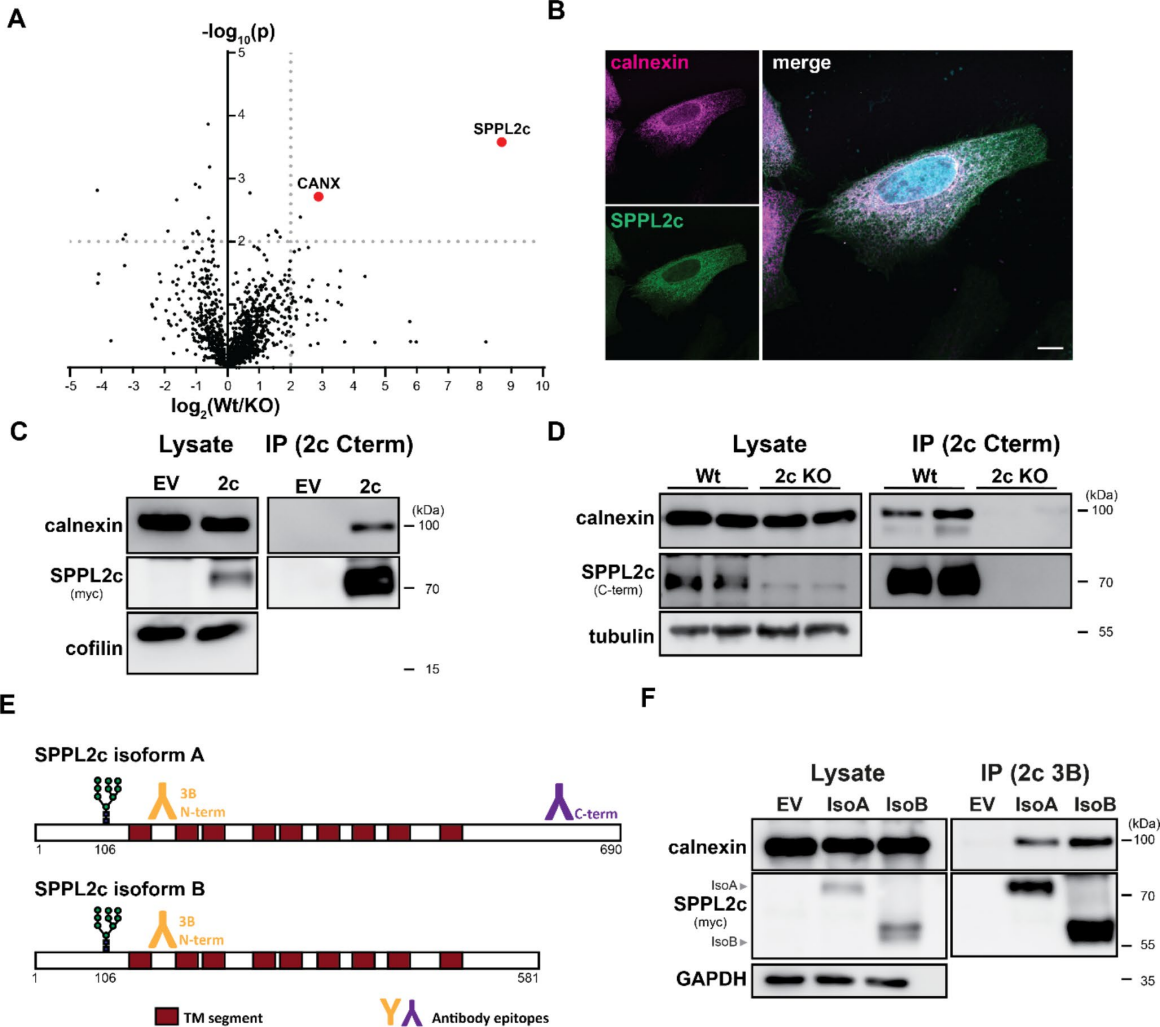
Calnexin is a novel binding partner of SPPL2c. (**A**) Binding partner of SPPL2c were identified by an unbiased IP-MS approach. SPPL2c was precipitated from testis lysates of either wild type (Wt) or SPPL2c-deficient (KO) mice using a SPPL2c-targeting antibody coupled to sepharose beads. Bead eluates were analysed mass spectrometrically. Hits identified in both independent runs that were significantly enriched in the Wt fractions are highlighted in the presented Volcano plot. *N* = 2, *n* = 4. (**B**) HeLa cells transiently transfected with SPPL2c-myc were fixed and stained with either anti-myc or anti-calnexin. Scale bar, 10 μm. (**C**) HEK293T cells were transiently transfected with SPPL2c-myc or an empty vector (EV) control. SPPL2c was precipitated from cell lysates with anti-SPPL2c beads and levels of the indicated proteins in total lysates as well as bead eluates (IP) were evaluated by western Blotting. (**D**) Testis lysates from either Wt or SPPL2c-deficient mice were subjected to immunoprecipitation analysis employing anti-SPPL2c beads. SPPL2c-containing complexes were subsequently analysed by western blotting. (**E**) Scheme of the two murine SPPL2c isoforms highlighting the SPPL2c-targeting antibodies employed in this study. (**F**) Binding of calnexin to the two different SPPL2c isoforms was monitored by co-immunprecipitation of SPPL2c complexes from transiently transfected HEK293T cells lysed in presence of 0.5% DDM. For precipitation, an antibody recognising both isoforms (3B epitope) coupled to sepharose beads was employed

Having identified Frey1 as novel regulator of SPPL2c that at least in cell-based overexpression systems efficiently blocks the proteolytic activity of this enzyme in a highly specific manner [21, 22], we hypothesised that SPPL2c might also be subject to regulation by other proteins. To characterise the in vivo interactome of this intramembrane protease, we aimed to specifically isolate SPPL2c-containing protein complexes from the murine testis and subject them to mass spectrometric analysis employing our specific antibody targeting the C-terminus of the protease. For this purpose, we first determined the optimal detergent for SPPL2c complex isolation. Testes of either wild type (Wt) or *Sppl2c*^−/−^ mice were lysed in presence of either 0.5% CHAPSO, DDM, DM or digitonin and SPPL2c was precipitated using anti-SPPL2c beads. Bead eluates (IP fraction) as well as supernatants (unbound fraction) were subsequently analysed by western blotting for the presence of SPPL2c as well as its established interactor Frey1 as positive control. As depicted in Suppl. Fig S1, even though similar to digitonin, precipitation of SPPL2c as well as co-purification of Frey1 (Suppl. Fig. S1A) and depletion of SPPL2c from total lysates (Suppl. Fig. S1B) was most efficient in the presence of 0.5% DDM which therefore was selected for the following IP-MS approach. Mass spectrometric analysis of SPPL2c precipitates from total testis lysates revealed a significant enrichment of SPPL2c in wild type samples if compared to the corresponding knockout controls validating the general experimental approach (Fig. 1A). However, unfortunately in none of the two independent experiments (four mice per genotype in total) Frey1, that could be readily detected in immunoprecipitates of SPPL2c by Western Blotting (Fig. S1A), was identified by the employed IP-MS approach. This might be related to the small size of the protein. Beyond SPPL2c, the only candidate that was significantly co-purified with the protease was calnexin, that was approximately 7-fold more abundant in wild type precipitates than in the corresponding knockout controls. Calnexin is a type I transmembrane protein representing the major ER-resident chaperone involved in protein quality control. Binding of misfolded proteins by calnexin typically occurs in a lectin-like manner and depends on the presence of monoglycosylated glycans in the client protein. In a non-canonical mode, calnexin also detects misfolded transmembrane domains of membrane-embedded proteins to ensure the correct folding of these molecules [27, 28].

Based on the rather promiscuous binding of calnexin to a large number of misfolded proteins, we first aimed to validate its observed interaction with SPPL2c. As expected, calnexin co-localised with overexpressed SPPL2c-myc in the ER of transfected HeLa cells (Fig. 1B), representing an essential, even though not sufficient criterion for interaction. Furthermore, endogenous calnexin was co-purified with SPPL2c from lysates of transiently transfected HEK293T cells (Fig. 1C). Most importantly, calnexin was also specifically detected in bead eluates upon precipitation of SPPL2c from wild type, but not *Sppl2c*^−/−^ testes (Fig. 1D), validating the observed interaction at the full endogenous level in the murine testis.

Within the murine testis, SPPL2c exists in two functionally redundant isoforms (isoform A and B), that only differ in the length of their cytosolic C-termini (Fig. 1E) [5]. Since so far all precipitation experiments were carried out with the longer A isoform or employing an antibody specifically targeting the C-terminus of isoform A sparing the smaller B isoform, we next analysed whether recruitment of calnexin was specific for isoform A or a common feature of all known SPPL2c variants. Again, HEK293T cells were transfected with expression vectors encoding either SPPL2c isoform A-myc or SPPL2c isoform B-myc. However, this time SPPL2c was precipitated employing the 3B antibody targeting the loop between transmembrane segment 1 and 2 of SPPL2c that efficiently detects both isoforms (schematic representation in Fig. 1E). As depicted in Fig. 1F, in addition to isoform A calnexin also clearly interacted with the smaller B isoform suggesting that recruitment of calnexin does not represent a privilege of the full length SPPL2c isoform and therefore also does not require the C-terminus of the intramembrane protease.

**Fig. 2:**
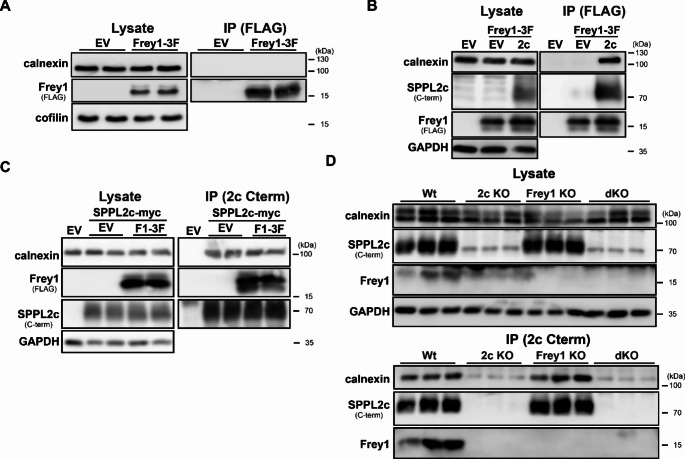
Calnexin recruitment to SPPL2c is not affected by Frey1. (**A**) HEK293T cells were transiently transfected with Frey1-3xFLAG (Frey1-3xF) or an empty vector (EV) control and the small type II protein was subsequently precipitated from total cell lysates using anti-FLAG beads. Bead eluates (IP) as well as total lysates were finally analysed by western blotting. (**B**) Same as in A), but cells were additionally co-transfected with SPPL2c-myc (2c) as indicated. (**C**) HEK293T cells were transiently transfected as described in B), but this time SPPL2c-myc was precipitated using anti-SPPL2c beads. (**D**) Testes from either wild type (Wt), SPPL2c- (2c KO), Frey1- (Frey1 KO) or double-deficient (dKO) mice were lysed with 0.5% DDM and SPPL2c was pulled down with an antibody targeting its C-terminus coupled to sepharose beads. Presence of the indicated proteins in IP samples and total lysates was monitored by western blotting

As demonstrated before, SPPL2c interacts with the small testis-specific type II protein Frey1 that in cell-based overexpression systems is able to block SPPL2c activity by binding to its catalytic centre. Even though expression of Frey1 was not likely to be required for the SPPL2c-calnexin interaction since this protein is not expressed at the endogenous level in HEK293T cells, we were interested whether presence of the small type II protein would affect the formation of SPPL2c-calnexin complexes, also since it has been suggested to interact with various other ER chaperones [24]. In a first approach, we tested whether Frey1 could interact with calnexin independent of the intramembrane protease. As depicted in Fig. 2A and in agreement with the absence of an intrinsic glycosylation site in Frey1, calnexin was not co-purified with overexpressed Frey1-3xFLAG in transfected HEK293T. By contrast, co-expression of SPPL2c-myc with Frey1-3xFLAG facilitated the detection of the ER chaperone in Frey1-3xFLAG immunoprecipitates (Fig. 2B), suggesting that Frey1 and calnexin do not physically interact but can be recruited independently to SPPL2c occupying different binding sites. Next, we aimed to analyse whether binding of Frey1 would affect the incorporation of calnexin into SPPL2c complexes. For this purpose, we employed the same experimental setup as described in Fig. 2B, but pulled down SPPL2c-myc instead of Frey1-3xFLAG. Intriguingly, the amount of calnexin in SPPL2c complexes was not modulated by the presence of the SPPL2c inhibitory protein (Fig. 2C), strongly arguing for a non-competitive binding mode. This was further validated by the analysis of testicular SPPL2c complexes from wild type, *Sppl2c*^−/−^, *Frey1*^−/−^ and *Frey1*^−/−^*Sppl2c*^−/−^ mice. As depicted in Fig. 2D and in agreement with the data presented in Fig. 1D, calnexin was exclusively detected in anti-SPPL2c bead eluates in presence of the protease. Moreover, levels of co-purified calnexin did not differ between wild type and *Frey1*^−/−^ mice, arguing against a competitive binding mode because loss of the other SPPL2c interactor did not facilitate increased recruitment of the ER chaperone to the intramembrane protease.

**Fig. 3:**
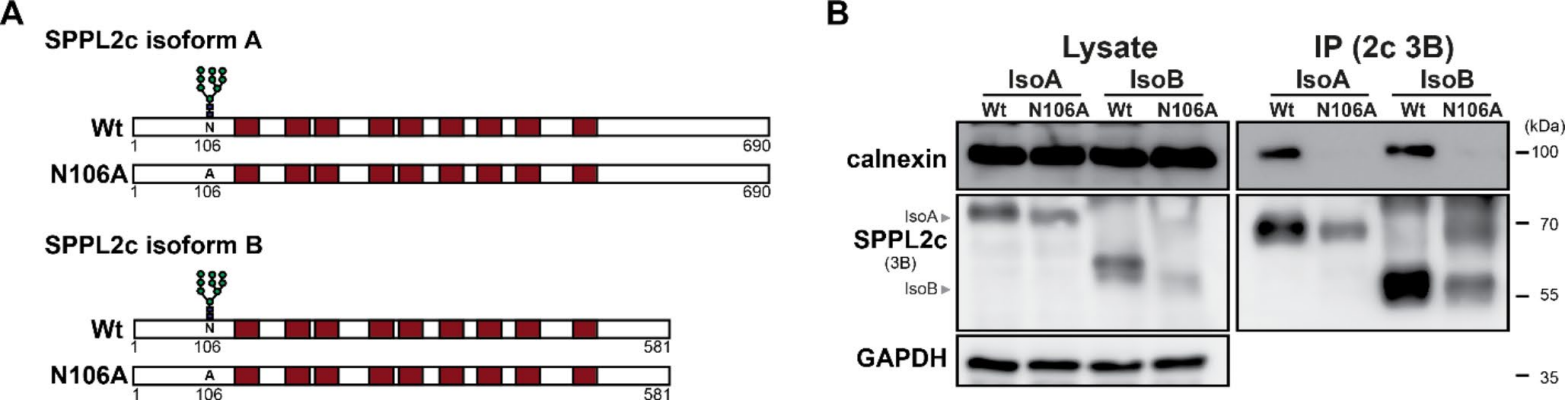
N-glycosylation of SPPL2c is essential for interaction with calnexin. (**A**) Scheme of murine SPPL2c isoforms A and B, showing the single glycosylation site at N106 of both isoforms. Transmembrane domains are indicated as coloured boxes. (**B**) HEK293T cells transiently transfected with either wild type (Wt) or glycosylation-deficient (N106A) SPPL2c-myc variants of isoform A and B of the protease were lysed in 0.5% DDM. SPPL2c was subsequently isolated using an antibody recognising both isoforms coupled to sepharose beads. Interaction of all SPPL2c variants with calnexin was finally evaluated by western blotting

Since binding sites of Frey1 and calnexin in SPPL2c were most likely not identical, we next aimed to identify the mode of interaction between the ER chaperone and the protease. Calnexin either binds client proteins based on the presence of luminal monoglucosylated glycans employing its lectin domain or recognises misfolded transmembrane domains by specific residues within its own transmembrane segment. SPPL2c contains a single N-glycosylation site within its luminal N-terminus that is present in both isoforms (Fig. 1E). In both cases, this site is also used at the endogenous level in the murine testis as demonstrated previously by employing endoglycosidase H deglycosylation experiments [5]. Since these N-terminal glycosylation sites resembled likely targets for calnexin, we made use of previously generated mutants of both isoforms in which the asparagine at position 106 was mutated to an alanine and which therefore are not glycosylated anymore (Fig. 3A) [5]. HEK293T cells were transfected with either the wild type or N106A mutants of both myc-tagged isoforms of SPPL2c and SPPL2c was isolated using anti-SPPL2c 3B-beads detecting both SPPL2c variants. Mutation of N106 caused a slightly increased mobility of both SPPL2c isoforms in SDS-PAGE validating the loss of glycosylation at this position (Fig. 3B). Intriguingly, binding of calnexin was abolished by the N106A mutation for both isoforms, highlighting the essential function of the N-glycosylation of SPPL2c for the formation of the SPPL2c-calnexin complex, which is in line with the canonical lectin-like binding activity of the ER chaperone.

**Fig. 4:**
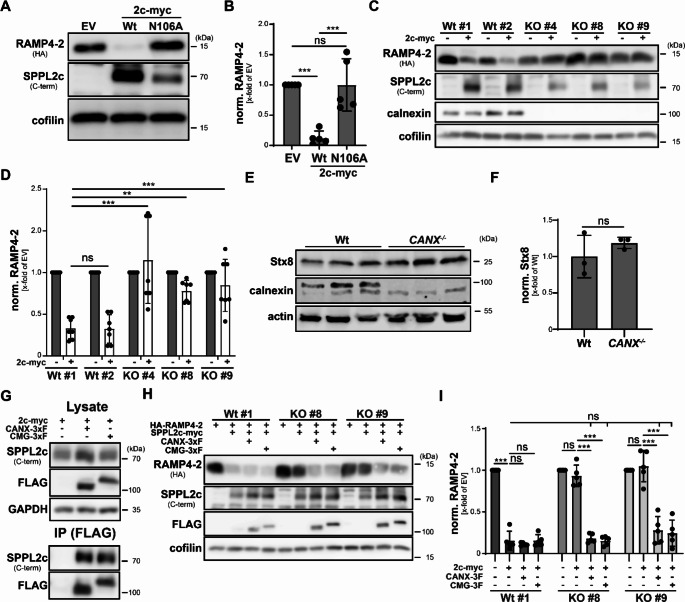
Calnexin is required for the proteolytic activity of SPPL2c in cell-based systems. (**A**) HEK293T cells were transfected with HA-RAMP4-2 as model substrate and either an empty vector control (EV) or wild type (Wt) or glycosylation-deficient (N106A) SPPL2c-myc (2c-myc). Processing of HA-RAMP4-2 by the SPPL2c variants was analysed by western blotting upon cell lysis. (**B**) Quantification of A). *N* = 5, *n* = 5. One-Way ANOVA with Tukey’s post hoc testing. (**C**) Wild type or calnexin-deficient HEK293T cells were transfected with HA-RAMP4-2 and SPPL2c-myc as indicated. Cleavage activity of SPPL2c was again monitored by western blotting. (**D**) Quantification of C). *N* = 7, *n* = 7.Two-Way ANOVA with Tukey’s multiple comparisons test. (**E**) Protein amounts of the established SPPL2c substrate Syntaxin-8 (Stx8) in total lysates of testes of either Wt or calnexin-deficient mice were evaluated by western blotting. (**F**) Quantification of E). *N* = 1, *n* = 3. Unpaired two-tailed Student’s t-test. (**G**) HEK293T cells were transiently transfected with SPPL2c-myc alone or together with either 3xFLAG-tagged calnexin (CANX-3 F) or calmegin (CMG-3 F). Upon cell lysis in presence of 0.5% DDM and precipitation of the ER chaperones with anti-FLAG beads, the indicated proteins were analysed by Immunoblotting in total lysates and bead eluates (IP). (**H**) Wild type (clone #1) or calnexin-deficient (clone #8 and #9) HEK293T cells were transfected with HA-RAMP4-2 alone or together with 2c-myc. Where indicated, cells were additionally incubated with plasmid DNA encoding either CANX-3 F or CMG-3 F. Processing of HA-RAMP4-2 in the different cell lines was subsequently monitored by western blotting. (**I**) Quantification of H). *N* = 5, *n* = 5. Two-Way ANOVA with Tukey’s post hoc test. ns, not significant; ** *p* ≤ 0.01; *** *p* ≤ 0.001

Following the validation of the SPPL2c-calnexin interaction as well as the identification of the glycosylation site at N106 of the intramembrane protease as an essential binding site, we aimed to delineate the functional consequences of the observed interaction. We first characterised the impact of calnexin on the catalytic activity of the enzyme. Since so far both isoforms of SPPL2c behaved the same with regard to recruitment of calnexin, we proceeded with the full length A isoform for further analysis. In a first approach, we compared the proteolytic activity of the afore-mentioned SPPL2c N106A mutant that is incapable of calnexin binding with that of the corresponding wild type protein, employing established cell-based protease assays. HEK293T cells were either transfected with the SPPL2c substrate HA-RAMP4-2 alone or together with wild type SPPL2c-myc or its N106A mutant. Substrate processing was subsequently analysed by western blotting. While co-expression of wild type SPPL2c-myc significantly reduced HA-RAMP4-2 levels, its N106A mutant, which however was less abundant than the wild type SPPL2c protein, failed to proteolyse the substrate (Fig. 4A, B). This was still observed in cells with equal expression of wild type and mutated SPPL2c (Suppl. Fig. S2A, B) achieved by transfection of HEK cells with a fourfold increased amount of 2c N106A-encoding plasmid if compared to the wild type construct, demonstrating that indeed non-glycosylated SPPL2c lacks catalytic activity. This effect was not specific for HA-RAMP4-2 but also applied to other SPPL2c substrates like HA-Syntaxin-8 (Suppl. Fig. S2C, D) and 3xFLAG-phospholamban (Suppl. Fig. S2E, F), that were analysed by similar cleavage assays. We additionally monitored SPPL2c-mediated processing of HA-HO-1 by indirect immunofluorescence. Processing of most SPPL2c substrates leads to the generation of a rather unstable intracellular domain that in many cases cannot be detected by Immunoblotting. By contrast, intramembrane proteolysis of HO-1 by SPPL2c generates a rather stable HO-1 intracellular domain (ICD), which is not anchored to the ER but released to the cytosol and even translocates to the nucleus. This can be assessed by indirect immunofluorescence as experimental readout for proteolytic processing of the tail-anchored protein. Upon transfection of HeLa cells the full-length HA-HO-1 protein was predominantly detected in a reticular ER-like membrane network (Suppl. Fig. S2G, H). Co-expression of wild type SPPL2c-myc caused a redistribution of HA-HO-1 staining to the cytosol and the nucleus resulting from the proteolytic release of the HA-HO-1 ICD. Comparable to the results obtained for the other tested substrates, cytosolic release and nuclear translocation of the HA-HO-1 was not observed following co-transfection with either catalytically inactive SPPL2c D/A or the N106A mutant highlighting the relevance of the N-glycosylation site for proteolytic activity of SPPL2c.

Interfering with N-glycosylation of proteins can have multiple side effects. To pinpoint that the loss of proteolytic activity of SPPL2c N106A was indeed caused by its inability to bind calnexin, we wanted to validate the findings employing a genetic approach. For this purpose, we generated calnexin-deficient HEK293T cells employing CRISPR/Cas9 technology specifically targeting exon 5 and 7 of the human *CANX* gene with three different sgRNAs. After subcloning of the transfected cell line, knockout of the *CANX* gene was validated by western blotting (Suppl. Fig. S3A). Three independent KO clones (#4, #8, #9) and a single calnexin-positive clone (#1) were selected for further analysis together with the parental wild type cell line. According to previous cleavage assays, cells were transfected with either HA-RAMP4-2 alone or together with wild type SPPL2c-myc. As a readout, HA-RAMP4-2 levels were visualised by western blotting. As expected, co-transfection with wild type SPPL2c-myc reduced HA-RAMP4-2 levels in both the parental as well as the subcloned wild type cell lines (Fig. 4C, D). By contrast and despite only mildly reduced expression levels of SPPL2c if compared to the wild type cells, loss of the calnexin protein completely blocked SPPL2c-dependent HA-RAMP4-2 processing in all three tested knockout clones, demonstrating the crucial relevance of calnexin for the proteolytic activity of SPPL2c within this system. We next monitored whether loss of calnexin also affected the proteolytic activity of SPPL2c in a physiological context. For this purpose, we lysed testes from either wild type or calnexin-deficient mice and analysed the expression levels of Stx8, an established in vivo substrate of SPPL2c, by western blotting. As depicted in Fig. 4E and quantified in 4 F, loss of calnexin did not result in a major alteration in Stx8 protein amounts, even though they significantly accumulated in testes of SPPL2c-deficient mice (Suppl. Figure S3B, C). This suggested that the loss of calnexin with regard to SPPL2c activity might be compensated under physiological conditions. A promising candidate protein for this is calmegin, a testis-specific calnexin homologue essential for male fertility [29–31]. To collect evidence for a potential compensatory mechanism, we cloned C-terminally 3xFLAG-tagged versions of calnexin and calmegin from testis cDNA and compared them for their impact on SPPL2c. As depicted in Fig. 4G, SPPL2c-myc was efficiently co-purified with both calnexin-3xFLAG and calmegin-3xFLAG from lysates of transiently transfected HEK293T cells, indicating that calmegin at least under the employed conditions can interact with the protease. In addition, co-expression of calmegin was able to restore the catalytic activity of SPPL2c in calnexin-deficient cells to a similar extent as calnexin itself (Fig. 4H, I), indicating that both homologues impact on SPPL2c in similar ways and therefore also under endogenous conditions could compensate for each other.

**Fig. 5:**
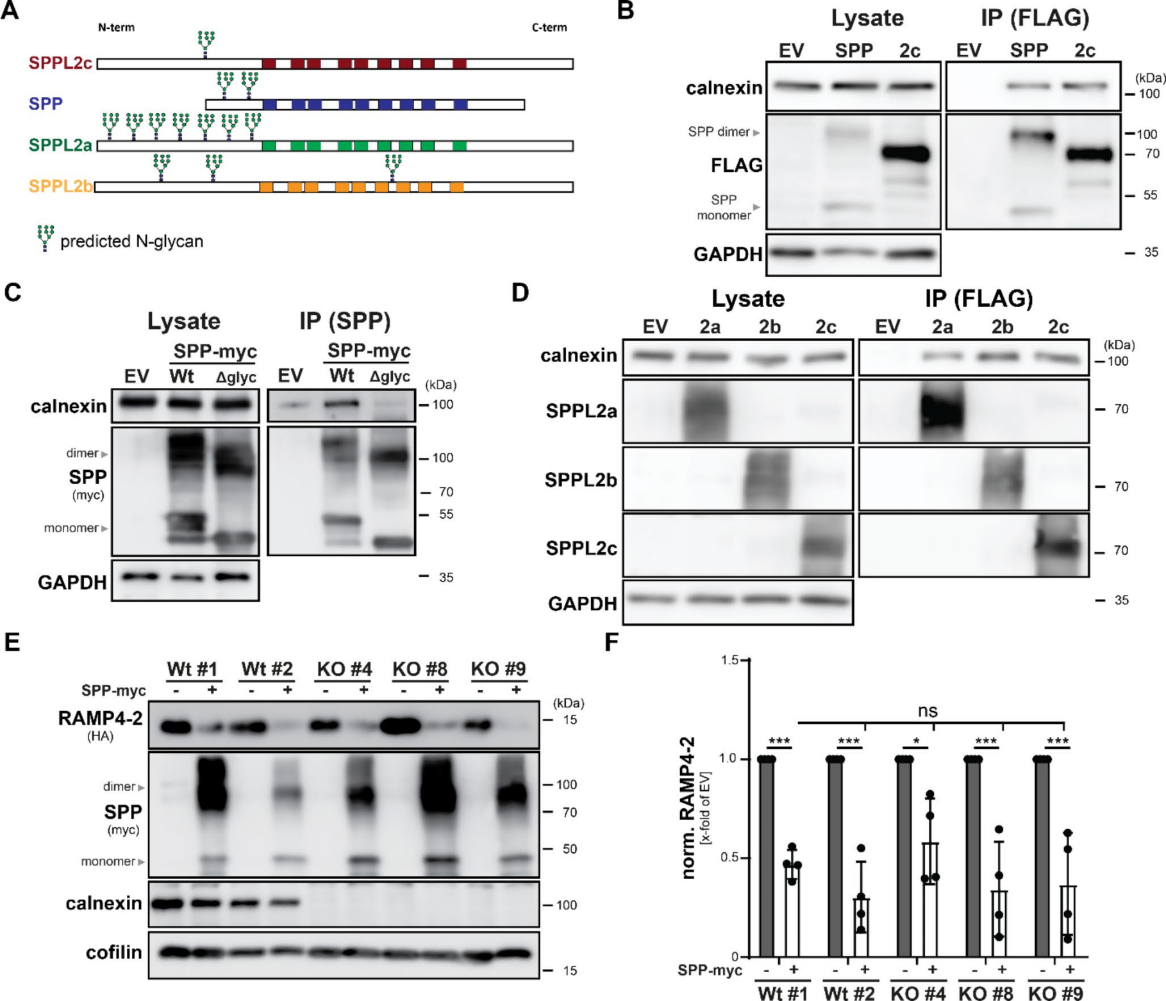
Calnexin specifically regulates SPPL2c-mediated intramembrane proteolysis. (**A**) Schematic representation of SPP and the three SPPL2 proteases highlighting the predicted glycosylation sites of the enzymes. Transmembrane segments are depicted as coloured boxes. (**B**) HEK293T cells were transiently transfected with 3xFLAG-tagged versions of SPP and SPPL2c (2c) or an empty vector (EV) control and both proteases were purified with anti-FLAG beads from total lysates. Interaction with calnexin was monitored by western blotting. (**C**) Following transient transfection with either wild type (Wt) or glycosylation-deficient (N10/20A, Δglyc) SPP-myc, HEK293T cells were lysed with 0.5% DDM and SPP variants were precipitated using an antibody targeting the C-terminus of the protease coupled to sepharose beads. Bead eluates (IP) as well as total lysates were analysed by western blotting. (**D**) The experiment described in B) was repeated employing SPPL2a-3xFLAG (2a), SPPL2b-3xFLAG (2b) and SPPL2c-3xFLAG (2c) constructs. (**E**) Processing of HA-RAMP4-2 by co-expressed SPP-myc was monitored by Western blotting of total lysates of either Wt or calnexin-deficient HEK293T cells upon transient transfection. (**F**) Quantification of E). *N* = 4, *n* = 4. Two-Way ANOVA with Tukey’s multiple comparisons test. ns, not significant

We wondered whether the strong relevance of calnexin for SPPL2c activity in cell-based systems would also apply to other SPP/SPPL proteases. With exception of SPPL3, all other family members carry N-glycosylations in their N-termini (Fig. 5A) comparable to SPPL2c. This question was especially relevant, since the ER also harbours a second aspartyl intramembrane protease, SPP, which comparable to SPPL2c is also expressed in high amounts in the murine testis, even though in different high molecular weight complexes [5]. We first transfected HEK293T cells either with SPP-3xFLAG or SPPL2c-3xFLAG as positive control and isolated protease-containing complexes using anti-FLAG beads after cell lysis. Under these conditions, calnexin could be detected both in bead eluates of SPPL2c and SPP transfected cells, indicating that the chaperone also binds to the other ER-resident SPP/SPPL family member (Fig. 5B). SPP carries two potential N-glycosylation sites within its luminal N-terminus, which based on the findings for SPPL2c represented the most probable interaction site. To validate the binding of calnexin to the N-glycosylation sites in murine SPP, we simultaneously mutated N10 and N20 to alanines. Replacement of these asparagine residues led to a clear reduction of the apparent molecular weight of SPP upon expression in HEK293T cells (Fig. 5C) validating the use of the two sites in the applied system. Comparable to SPPL2c, ablation of the N-glycosylation of SPP abolished co-purification of calnexin upon precipitation of the protease to background levels, proving the relevance of the mutated residues for the assembly of the SPP/calnexin complex. We also assessed interaction of calnexin with the two other glycosylated SPP/SPPL family members, SPPL2a and SPPL2b. By contrast to SPP and SPPL2c, under endogenous conditions these proteins predominantly localise to the endo-lysosomal compartment or the plasma membrane, respectively. However, on their way through the secretory pathway they have to pass the ER which enables at least transient contact with calnexin. As depicted in Fig. 5D, calnexin could be isolated together SPPL2b-3xFLAG in comparable levels to SPPL2c-3xFLAG, and - even though slightly less pronounced - also with SPPL2a-3xFLAG.

This data pointed to a rather promiscuous binding of calnexin to all glycosylated members of the SPP/SPPL family. Nevertheless, we were interested whether also for these proteases association with calnexin was required for their proteolytic activity. Again, we first tested the cleaving ability of the glycosylation-deficient SPP-myc N/A mutant. Since HA-RAMP4-2 is also proteolysed by SPP, in this case we employed the same model substrate as for the analysis of SPPL2c activity. Surprisingly, when monitoring the activity of wild type and mutated SPP-myc in transiently transfected HEK293T cells, by contrast to SPPL2c, glycosylation-deficient SPP performed equally well in the degradation of HA-RAMP4-2 as the wild type protease (Suppl. Fig. S4A, B), even though the glycosylation was also essential for calnexin recruitment in this case (Fig. 5C). In line with these findings, SPP-mediated HA-RAMP4-2 proteolysis was not affected in all tested calnexin-deficient cell lines (Fig. 5E, F), suggesting that despite the ability of this protease to recruit calnexin, interaction with the chaperone in this case is no prerequisite for proteolytic activity. Similar results were obtained for SPPL2a and SPPL2b. In this case, activity was monitored by analysing cleavage efficiency of HA-VAMP2, a recently described substrate of both enzymes [19]. Again, loss of calnexin expression only mildly (SPPL2a, Suppl. Fig. S4C, D), or not at all (SPPL2b, Suppl. Fig. S4E, F) reduced activity of both enzymes, highlighting the high relevance of the chaperone for activity of specifically SPPL2c despite its promiscuous recruitment to all family members.

**Fig. 6:**
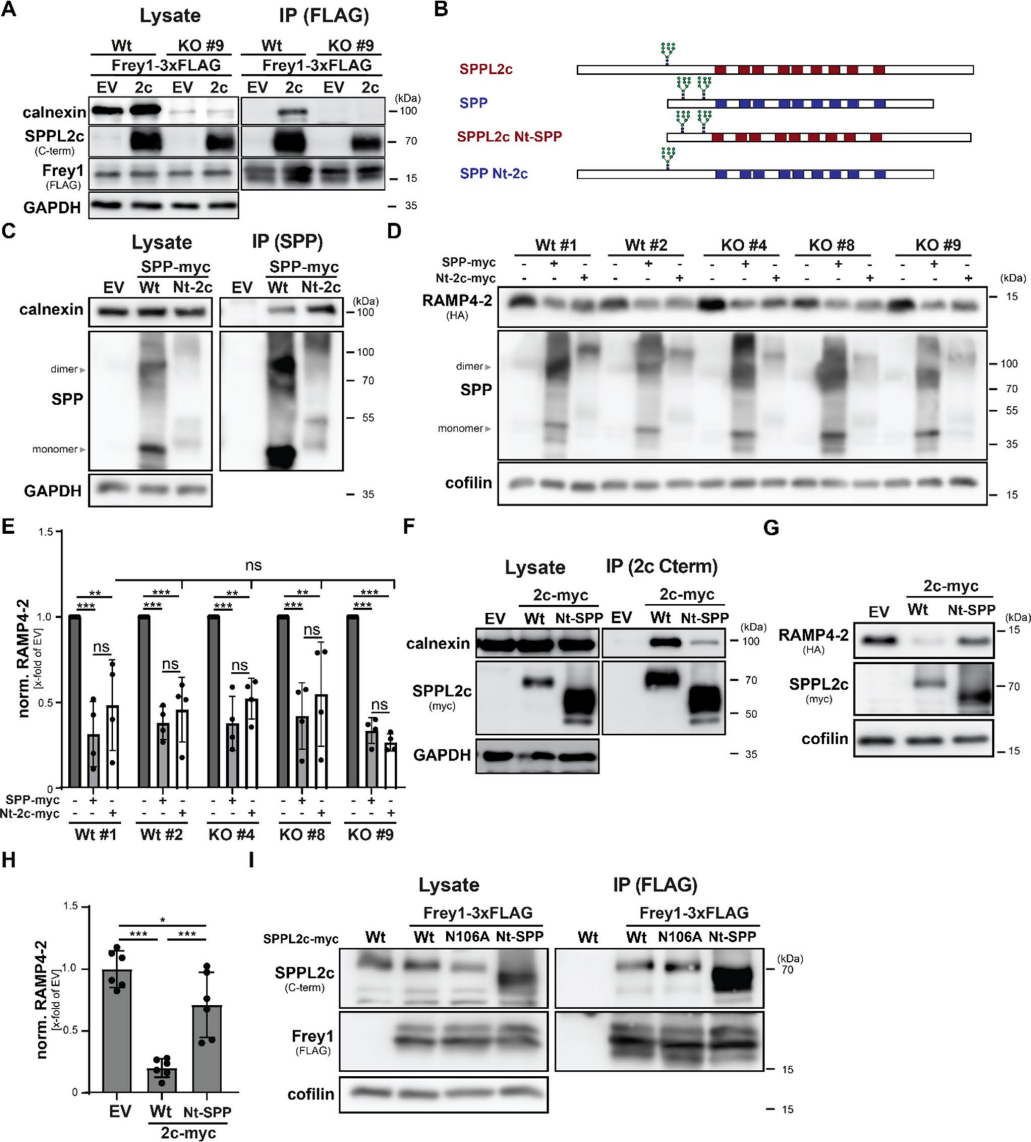
Misfolding of SPPL2c cannot fully explain the impact of calnexin on its protease activity. (**A**) Wild type (Wt) or calnexin knockout (KO) cells were transiently transfected with Frey1-3xFLAG either with an empty vector control (EV) or SPPL2c-myc following precipitation of Frey1 from DDM lysates employing anti-FLAG beads. Bead eluates (IP) as well as total lysates were finally analysed by western blotting. (**B**) Schematic representation of SPP-SPPL2c chimeras in which the N-termini of both proteases were swapped. Glycosylation sites as well as transmembrane segments (coloured boxes) are highlighted. (**C**) HEK293T cells were transfected with either Wt SPPL-myc or its Nt-2c mutant and the protease variants were precipitated from DDM lysates with anti-SPP beads. Co-purification of calnexin was finally monitored by western blotting. (**D**) Wt or calnexin-deficient HEK293T cells were transiently transfected with HA-RAMP4-2 and either SPP-myc or its Nt-2c chimera. Proteolytic turnover of HA-RAMP4-2 was assessed by immunoblotting. (**E**) Quantification of D). *N* = 4, *n* = 4. Two-Way ANOVA with Tukey’s multiple comparisons test. (**F**) Wild type SPPL2c-myc and its Nt-SPP chimera were precipitated from DDM lysates of transiently transfected HEK293T cells using anti-SPPL2c beads. Interaction of the protease variants with calnexin was evaluated by western blotting of total lysates as well as bead eluates. (**G**) The proteolytic activity of SPPL2c Nt-SPP-myc was compared to that of Wt SPPL2c-myc by co-expression with HA-RAMP4-2 in HEK293T cells. (**H**) Quantification of H). *N* = 3, *n* = 6. One-Way ANOVA with Tukey’s post hoc testing. (**I**) Different variants of SPPL2c-myc (Wt, N106A, Nt-SPP) were co-precipitated with Frey1-3xFLAG from total lysates of transiently transfected HEK293T cells using anti-FLAG beads. ns, not significant; * *p* ≤ 0.05; ** *p* ≤ 0.01; *** *p* ≤ 0.001

We wondered how despite the conserved binding of calnexin to all tested SPP/SPPL proteases the exclusive requirement of the ER chaperone for SPPL2c activity might be achieved at the molecular level. Since calnexin typically binds to misfolded proteins, we speculated that folding of SPPL2c might be impaired in the absence of the ER chaperone. This hypothesis was further substantiated by the notion that loss of calnexin or N-glycosylation of SPPL2c required for the recruitment of the chaperone led to a slightly decreased expression of the protease (Figs. 3B and 4A and C, Suppl. Fig. S2A, C). To obtain insights into differences in the overall architecture of SPPL2c in relation to calnexin expression, we made use of Frey1, another specific interactor of SPPL2c. The small type II protein binds to the catalytic centre of the protease, which requires the correct positioning of the two catalytic aspartates of the enzyme [22]. To monitor whether the overall architecture of SPPL2c and its active site might be affected by the loss of calnexin, we expressed Frey1-3xFLAG alone or together with SPPL2c-myc in either wild type or calnexin-deficient (clone #9) HEK293T cells and subsequently precipitated Frey1 from cell lysates using anti-FLAG beads. As shown in Fig. 6A, considering the slightly reduced expression levels of SPPL2c in the calnexin-deficient cells, SPPL2c-myc was co-purified with Frey1 in similar amounts in both wild type and *CANX*^−/−^ cells. Since this suggested a correct assembly of the membrane-embedded SPPL2c transmembrane fold, we next speculated that calnexin might be specifically required for the proper folding of the N-terminal protease-associated (PA) domain of SPPL2c that harbours the glycosylation site of the I-CLIP at N106 [32]. Intriguingly, SPP, whose activity was not affected by the loss of calnexin (Fig. 5), does not possess a similar structure within its short, however glycosylated N-terminus. Therefore, we generated myc-tagged chimeras between SPPL2c and SPP, in which the N-termini of both proteases were exchanged (SPPL2c Nt-SPP or SPP Nt-2c, Fig. 6B), and tested them for their proteolytic activity with regard to calnexin expression. If the PA domain of SPPL2c required calnexin for its proper 3-dimensional arrangement, its transfer to SPP should evoke an increased calnexin recruitment together with impaired proteolytic activity and degradation of the resulting chimera in absence of the chaperone. In line with this hypothesis, SPP Nt-2c indeed bound calnexin more efficiently than wild type SPP despite overall reduced abundance of this mutant (Fig. 6C). This was however not linked to a decreased proteolytic activity of the chimera which processed HA-RAMP4-2 in comparable amounts to wild type SPP (Fig. 6D, E). In contrast to SPPL2c, the catalytic activity of the chimeric protease was not compromised in absence of calnexin rather resembling features of the SPP wild type protein. Vice versa, the SPPL2c Nt-SPP chimera recruited calnexin much less efficiently than wild type SPPL2c despite increased protein levels (Fig. 6F). At the same time, the mutant presented a markedly reduced proteolytic capacity against HA-RAMP4-2 in cell-based cleavage assays (Fig. 6G, H) even in calnexin-expressing wild type HEK293T cells. Again, this was not attributed to a general misfolding of the membrane part of SPPL2c as judged from strong binding of the SPPL2c Nt-SPP chimera to Frey1 (Fig. 6I). Altogether, these findings strongly argue for additional and highly specific features within the SPPL2c molecule that require the most likely constitutive association of calnexin to acquire and maintain the catalytic activity of the enzyme.

## Discussion

The data presented here identify the ER chaperone calnexin as the first essential interactor of a SPP/SPPL protease in general. So far, only little is known about interactors of these enzymes. In addition to Frey1, which binds to the catalytic site of SPPL2c and acts as negative regulator of the protease, recently several tetraspanins including CD81 have been identified to either directly or indirectly associate with the related SPPL2a and SPPL2b proteases which can modulate their proteolytic activity in a protease-specific manner [33]. However, the identification of calnexin as essential interaction partner of SPPL2c is noteworthy, since despite the growing evidence for regulation of SPPL2 proteases in general these enzymes are still believed to be active without any additional co-factors. This notion was largely derived from the observation that overexpression of SPP/SPPL2 proteases in cell-based model systems led to a concomitant increase in the intramembrane proteolysis of established substrates of these enzymes [5, 16, 19, 34]. However, as demonstrated in this study, this does not exclude the interaction of SPP/SPPL proteases with highly abundant proteins whose expression is strong enough to not represent a rate-limiting bottleneck for SPP/SPPL activity. In this light, high calnexin levels within the HEK293T and HeLa cell lines employed for the analysis of SPPL2c-mediated intramembrane proteolysis might have masked the essential requirement of this chaperone for the catalytic activity of this enzyme.

While calnexin represents the first essential interaction partner of a SPP/SPPL2 protease, the finding that the proteolytic activity can be dependent on the availability of accessory proteins is not new for aspartyl I-CLIPs in general. Presenilin 1 and 2, the catalytically active subunits of the heterotetrameric γ-Secretase complex, require the presence of the three additional complex constituents Anterior pharynx-defective 1 (Aph1), Presenilin enhancer 2 (Pen-2) and nicastrin (Nct) [35]. Comparable to the SPPL2c-calnexin interaction, assembly of γ-Secretase complexes takes place in the ER [36]. The fully assembled complex is subsequently exported to various other subcellular compartments including the endosomal system but also the plasma membrane [37]. While Aph1, Pen-2 and Nct remain associated with the presenilins in a stable complex, it remains unclear whether calnexin binds SPPL2c in a transient manner or permanently interacts with the protease. By contrast to the γ-Secretase complex, SPPL2c remains localised within ER membranes [5] and therefore is constitutively accessible for the chaperone. In the canonical model of calnexin activity, binding of the chaperone to misfolded glycoproteins requires the presence of monoglucosylated mannose-rich sugars generated by the ER-resident glucosidases I and II. The release of the substrate molecules from calnexin represents the actual folding step and is accompanied by subsequent removal of the glucose moiety thereby preventing constitutive binding of the client protein to calnexin [38–40]. Since recruitment of calnexin to SPPL2c depends on the N-glycosylation of the protease (Fig. 3), it appears likely that also the observed SPPL2c-calnexin interaction is rather transiently required for the stabilisation of SPPL2c based on the chaperone activity of calnexin aiding the correct folding of the PA domain of the protease. However, there are several reasons to assume that calnexin rather represents an essential co-factor of SPPL2c constitutively associating with the enzyme. On the one hand, calnexin is readily co-purified in significant amounts even under conditions with physiological expression of both interaction partners. On the other hand, SPPL2a and SPPL2b also have complex PA domain structures in their N-termini [32]. Despite their at least transient interaction with calnexin on their route through the secretory pathway (Fig. 5D), the activity of both enzymes is not hampered by the absence of the chaperone (Suppl. Fig. S4). This points to largely non-redundant regulatory mechanisms between SPPL2 proteases, which is surprising considering similar structural features of these enzymes that even have partially overlapping substrate spectra at least upon overexpression [15, 16, 19, 26]. Finally, as demonstrated by the SPPL2c Nt-SPP chimera and titration assays with the non-glycosylated and calnexin binding-deficient N106A mutant of SPPL2c, decreased stability of SPPL2c in the absence of calnexin alone is not sufficient to explain its complete inactivity under these conditions. Despite an overall intact membrane fold as judged from efficient recruitment of Frey1 to its catalytic site and even increased stability if compared to wild type SPPL2c, this mutant almost completely fails to proteolyse SPPL2c substrates in cell-based assays (Fig. 6G, H). These points support our hypothesis that calnexin represents a constitutive co-factor of SPPL2c that keeps the protease in an active conformation rather than being a transient interaction partner of the enzyme. Even though the glycosylation of SPPL2c is essential for its interaction with calnexin, recent reports that calnexin can also recognise client proteins based on transmembrane domain features [28] might point to additional secondary interaction sites between both molecules. These might be masked by the strong impact of the glycosylation site on calnexin binding or not detectable using the detergents employed for co-immunoprecipitation. Structural data of the SPPL2c-calnexin complex will significantly aid our understanding of the interplay of both proteins.

Beyond these rather mechanistic considerations, the existence of SPPL2c-calnexin complexes in the murine testis poses the central question regarding the physiological function of this interaction. As highlighted in Fig. 4E, calnexin-deficient mice do not have significant alterations of the SPPL2c substrate Stx8 as determined by Western blot analysis of total testis extracts. This might be explained at least in part by the notion that loss of calnexin can be compensated by other chaperones including the testis-specific calnexin homologue calmegin. At least in cell-based assay systems, calmegin efficiently binds to SPPL2c and fully restores the proteolytic activity of the enzyme in absence of calnexin (Fig. 4G-I). Although both chaperones show large commonalities, including central biochemical properties regarding client recognition [41], they also differ in central biological aspects with respect to their testicular expression pattern and function in male fertility [29–31, 42]. Even though the experimental evidence for the redundant functions of calnexin and calmegin with regard to SPPL2c activity so far remains restricted to cell-based overexpression systems, it is tempting to speculate that the association of the protease with calmegin might also be responsible for unaltered SPPL2c substrate levels in mice lacking calnexin (Fig. 4). Future studies will need to unravel the relevance of calmegin for SPPL2c activity under physiological conditions.

Considering the function of the observed SPPL2c-calnexin but potentially also -calmegin interaction, one needs to consider that beyond its proteolytic activity SPPL2c also fulfills non-catalytic functions in the murine testis [21]. Under physiological conditions, large amounts of SPPL2c interact with the small type II protein Frey1, which resides within the catalytic site of the enzyme thereby blocking its activity [21, 22]. Intriguingly, similar to the situation observed for calnexin, the clear inhibitory potential of Frey1 on SPPL2c could not be recapitulated by altered substrate levels in Frey1-deficient mice [21], potentially due to functionally different pools of SPPL2c in the murine testis. While the inhibitory function of Frey1 on SPPL2c seems to be dispensable in vivo, the small type II protein is essential for male fertility by allowing the proper assembly of protein complexes required for sperm-oocyte fusion. In cell-based overexpression assays, this function of Frey1 is significantly augmented by SPPL2c, that acts as scaffold protein in this context [21]. Intriguingly, several of the sperm proteins like the central Izumo1 molecule but also SPACA6 are glycosylated and therefore might require ER chaperones as folding guards [43–45]. Along this line, ER stress strongly impacts on male fertility [46, 47]. Among the sperm proteins involved in gamete fusion, especially SPACA6 is sensitive to ER stress which might be causative for the loss of this protein in Frey1-deficient mice [24, 45, 48]. Given the observation that Frey1, a SPPL2c inhibitor, and calnexin, a SPPL2c activator, do not bind mutually exclusive but can be found in the same protein complexes (Fig. 2B), it is tempting to speculate that SPPL2c might also attract calnexin to nascent sperm fusion complexes thereby aiding the proper folding of critical fusion factors. The other way around, calnexin might also recruit SPPL2c to areas with increased concentrations of misfolded membrane proteins for their elimination. However, all currently identified substrates of SPPL2c are tail-anchored proteins with no or only little luminal domains. Typically, these proteins are non-glycosylated and therefore at least do not resemble typical clients of the chaperone. Nevertheless, since calnexin also recognises misfolded proteins based on transmembrane domain interactions [28] and in this model a direct physical contact with the misfolded SPPL2c substrates is not essential, calnexin might also support recruitment of SPPL2c to hot spots of membrane protein folding.

In summary, we identify calnexin as a novel, specific activator and co-factor of the testis-specific intramembrane protease SPPL2c. Our data does not only provide significant insights into the regulation of this unique enzyme, but also points to the urgent requirement to better understand the regulation of these versatile proteases under different (patho-)physiological conditions.

## Electronic supplementary material

Below is the link to the electronic supplementary material.

Supplementary Material 1

Supplementary Material 2

Supplementary Material 3

## Data Availability

The mass spectrometry proteomics data have been deposited to the ProteomeXchange Consortium via the PRIDE [49] partner repository with the dataset identifier PXD052965, member of the ProteomeXchange consortium [50]. All data are available upon request to the corresponding author.
