# Supplementary material for: Selective regulation of aspartyl intramembrane protease activity by calnexin
Source: Cell Mol Life Sci. 2024 Oct 26;81(1):441. doi: 10.1007/s00018-024-05478-8 (PMC11513070; doi:10.1007/s00018-024-05478-8)
Supplement: Supplementary file 1 — Supplementary Material 1 [file 18_2024_5478_MOESM1_ESM.pdf]

Instrument & Software Supplement Project TMR05/06

METHODS SUPPLEMENTARY TABLES 2

Instrumentation 2
Q-Exactive HF - DDA 2
Thermo Dionex3000 RSLC 3

Software 3
Software Versions 3
Mascot Parameter 3

METHODS SUPPLEMENTARY TABLES

INSTRUMENTATION

Q-EXACTIVE HF - DDA

Instrument / Parameter	Value	Comments
**Q-Exactive HF**	ThermoScientific, Bremen, Germany	**DDA-Mode (positive ion mode)**
**MS1**		
Polarity	positive	
Resolution	R120000 at m/z 200	
AGC	3x 10E6	
Max. Fill Time	100ms	
Lock Mass	m/z 445.120025	Dodecamethylcyclohexasiloxane *[1]*
Scan Range	m/z 395-1500	
Picotip Needle	20µm / 10µm	NewObjectives, Ithaca, USA
Voltage	2.3-2.7kV	(might vary between experiments)
**MS2 High Res**	Top10	**HCD**
Resolution	R15000 at m/z 200	
AGC	1E5	
Max. Fill Time	50ms	
Isolation	2.0 m/z	
Isolation window Offset	0.3 m/z	
Scan Range	200-2000 m/z	
Fixed 1st Mass	-	
Norm. Collision Energy	27	
Threshold	2E3 / 4E4	
Charge states	Unassigned, 1, 6-8, >8	(rejected)
Dynamic Exclusion	15s / 3ppm	

THERMO DIONEX3000 RSLC

Instrument / Material	Manufacturer (Supplier)	Comments
**Dionex3000 RSLC**	ThermoScientific, Idstein, Germany	Nanoflow System
Acclaim PepMap 100 C18,<br>3 µm, 300 µm x 5 mm,<br>Acclaim PepMap C18 3,<br>µm, 75 µm x 15 cm	ThermoScientific, Idstein, Germany	Trap-Column Setup<br>Load: 2µl/min<br>Separation: 200nl/min
Picotip Needle 20 µm / 10 µm	NewObjectives, Woburn, USA	

SOFTWARE

SOFTWARE VERSIONS

**Instrument / Material**	Manufacturer (Supplier)	Comments
MASCOT V2.6 [2]	MatrixScience, London, UK	Protein Identification Software matrixscience.com
Progenesis QIP V4.2	Nonlinear Dynamics, Newcastle u.T., UK	Quantitative data interpretation (Peak Picking, MS/MS Export, Peptide ID import and Assignment, Quantification (MI3/Hi3))
Perseus 2.10	MPI for Biochemistry, Munich, DE	Data Aggregation

MASCOT PARAMETER

**Mascot Parameter QE-HF**	Value	Comments
**Version**	2.6	
MS Tolerance	10 ppm	
Protease	Trypsin	
Missed Cleavages	3	
Fixed Modifications	-	
Variable Modifications	Acetyl- N-Protein, Oxidation (M)	
MS/MS Tolerance	30 mmu	
Instrument	ESI-Quad	
Databases	Contaminants<br>Enzymes<br>STDs_TAGs	Databases applied according to sample origin.
Decoy	Yes	
