# Supplementary material for: Selective regulation of aspartyl intramembrane protease activity by calnexin
Source: Cell Mol Life Sci. 2024 Oct 26;81(1):441. doi: 10.1007/s00018-024-05478-8 (PMC11513070; doi:10.1007/s00018-024-05478-8)
Supplement: Supplementary file 2 — Supplementary Material 2 [file 18_2024_5478_MOESM2_ESM.pdf]

Supplementary Tables

oligos for CRISPR/Cas-mediated *CANX* knockout

sgRNAs

oligo ID	sequence (5' to 3')	targeted exon
**sgCANX-1**	tgtgttgtcttgtaggaaag	5
**sgCANX-2**	agcagagatggcatgatgct	5
**sgCANX-3**	cttcatagatacccgttttg	7

primers

primer ID	sequence (5' to 3')
**CANX-1_fwd**	cacctgtgttgtcttgtaggaaag
**CANX-1_rev**	aaacctttcctacaagacaacaca
**CANX-2_fwd**	caccagcagagatggcatgatgct
**CANX-2_rev**	aaacagcatcatgccatctctgct
**CANX-3_fwd**	caccсttcatagatacccgttttg
**CANX-3_rev**	aaaccaaaacgggtatctatgaag
**CANX_ex5_fwd**	tcaccgcaacctctgtttct
**CANX_ex5_rev**	acccaaaaatccctaccacca
**CANX_ex5_seq**	cacggctgccccagtataat
**CANX_ex7_fwd**	tcagttccatgacaagacccc
**CANX_ex7_rev**	ttgagtctcactttcgccca
**CANX_ex7_seq**	acagtgcccggccagatat
