# Supplementary material for: Selective regulation of aspartyl intramembrane protease activity by calnexin
Source: Cell Mol Life Sci. 2024 Oct 26;81(1):441. doi: 10.1007/s00018-024-05478-8 (PMC11513070; doi:10.1007/s00018-024-05478-8)
Supplement: Supplementary file 3 — Supplementary Material 3 [file 18_2024_5478_MOESM3_ESM.docx]

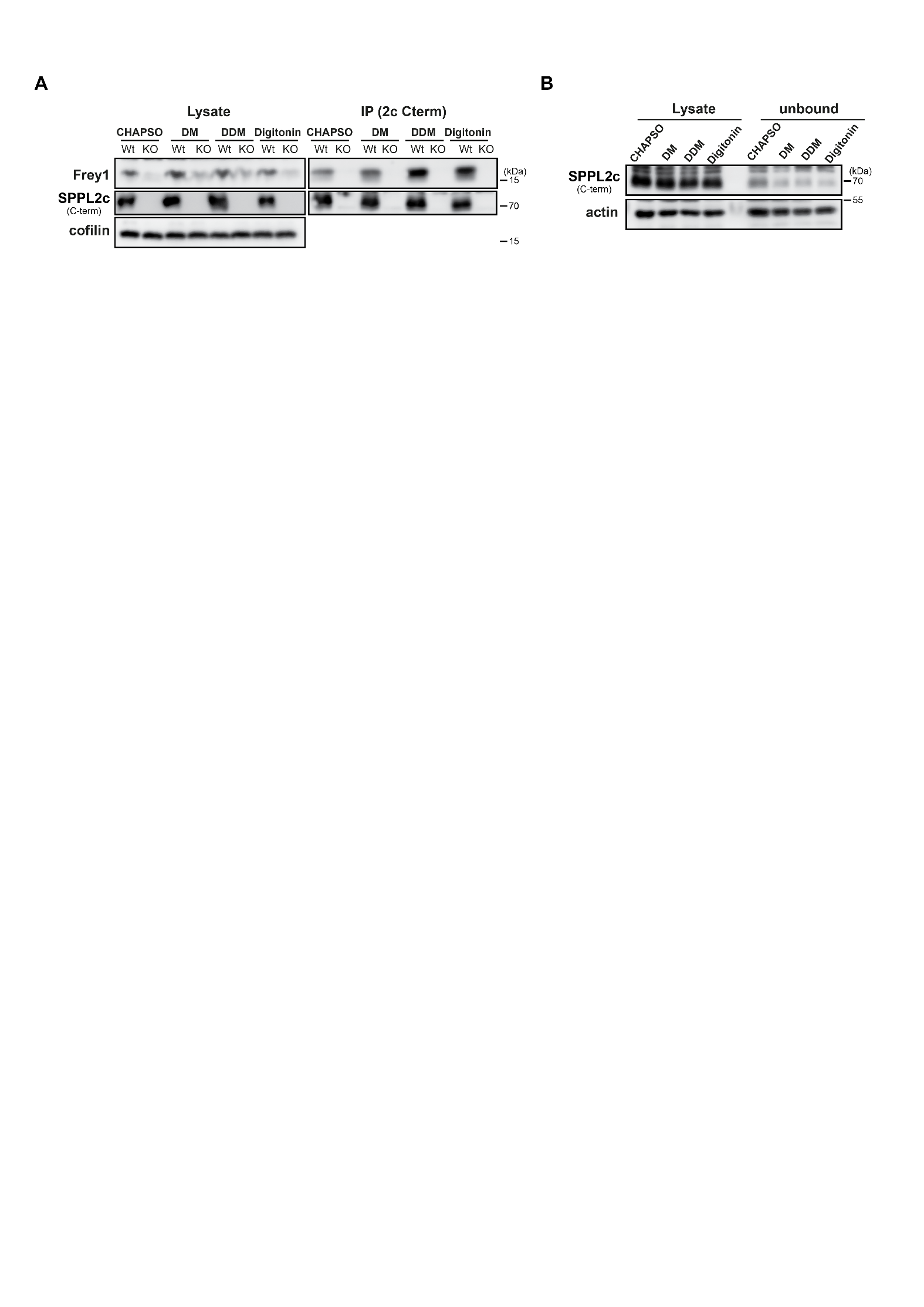


**Supplementary Figure 1. Optimisation of IP conditions for determination of the testicular interactome of SPPL2c.** Testes of either wild type (Wt) or SPPL2c-deficient (KO) mice were lysed in presence of either 0.5% DDM, 0.5% DM, 0.5% CHAPSO or 0.5% digitonin as indicated. SPPL2c-containing complexes were subsequently isolated employing an antibody against the C-terminus of the protease coupled to sepharose beads. Level of SPPL2c as well as its established interaction partner Frey1 were analysed in total lysates, bead eluates (IP) **(A)** and unbound fractions **(B)**.


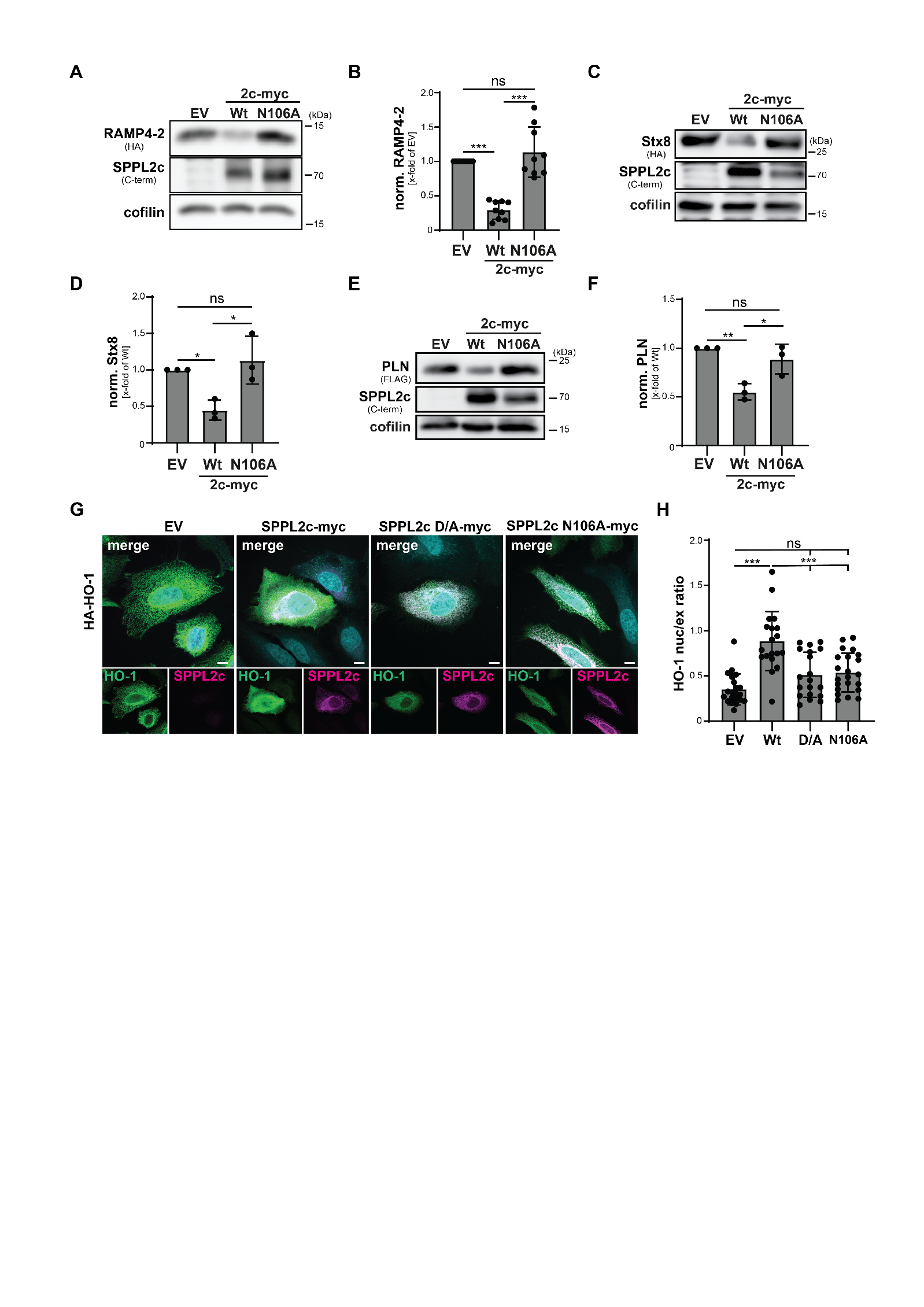


**Supplementary Figure 2. Glycosylation-deficient SPPL2c is catalytically inactive. A)** HEK293T cells were transfected with HA-Syntaxin-8 (Stx8) together with an empty vector (EV) control or either Wt or glycosylation-deficient (N106A) 2c-myc employing the respective plasmids at the same concentration. Processing of Stx8 was subsequently investigated by Immunoblotting. **B)** Quantification of A). N=3, n=3. One-Way ANOVA with Tukey’s post hoc testing. **C)** The experiment described in C) was repeated with 3xFLAG-PLN instead of HA-Stx8 as SPPL2c substrates. **D)** Quantification of C). N=3, n=3. One-Way ANOVA with Tukey’s post hoc testing. **E)** Following transient transfection of HeLa cells with HA-HO-1 together with either Wt SPPL2c-myc, its inactive D/A variant or its N106A mutant, cells were fixed with 4% PFA. The subcellular localisation of HA-HO-1 was evaluated by indirect immunofluorescence. Scale bars, 10 µm. **F)** Nuclear/extranuclear staining intensities of HO-1 were calculated for each experimental condition. N=3 (all), n=21(EV)/19(Wt, D/A)/22(N106A) cells per condition. One-Way ANOVA with Tukey’s post hoc testing. **G)** HEK293T cells were transiently transfected with HA-RAMP4-2 and either wild type 2c-myc or its non-glycosylated N106A mutant. To account for decreased expression of the N106A mutant upon transfection of identical DNA amounts, cells were incubated with a fourfold increased concentration of the 2c N106A encoding plasmid if compared to the wild type construct. Upon cell lysis, processing of HA-RAMP4-2 was analysed by Western blotting using the indicated antibodies. **H)** Quantification of G). N=9, n=9. One-Way ANOVA with Tukey’s post hoc testing. ns, not significant; * p≤0.05; ** p≤0.01; *** p≤0.001.


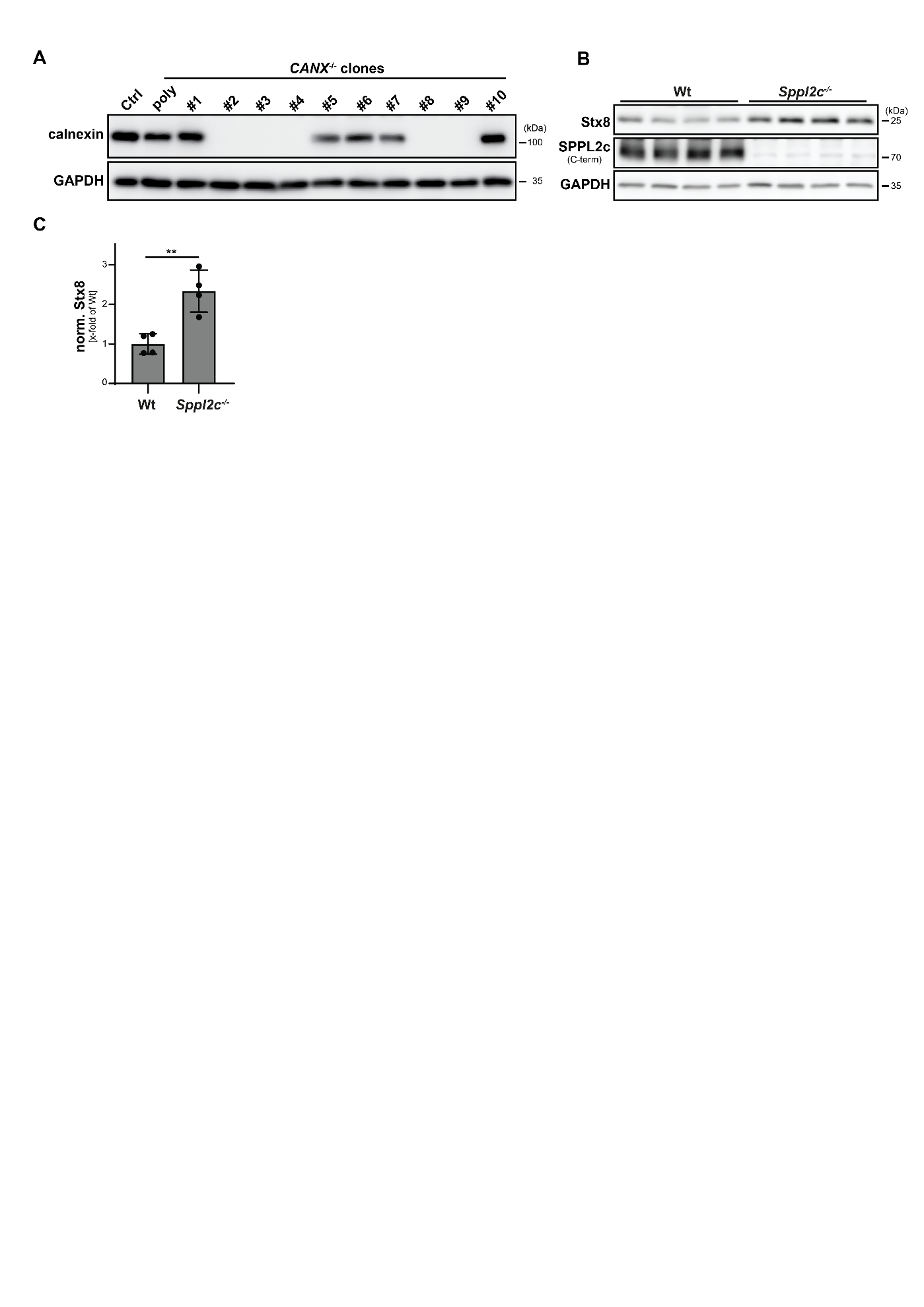


**Supplementary Figure 3. Calnexin is required for SPPL2c activity in cell-based assays.** **A)** Single cell clones derived from of HEK293T cells treated with a combination of three different sgRNAs targeting the calnexin locus as well as the parental wild type (Ctrl) and polyclonal (poly) cell batch prior to subcloning were analysed by Western blotting for their calnexin expression. **B)** Testes of either wild type (Wt) or SPPL2c-deficient mice were lysed and monitored for their syntaxin-8 (Stx8) protein levels by Western blotting. **C)** Quantification of B). N=1, n=4. Two-tailed unpaired Student’s t-test. ** p≤0.05


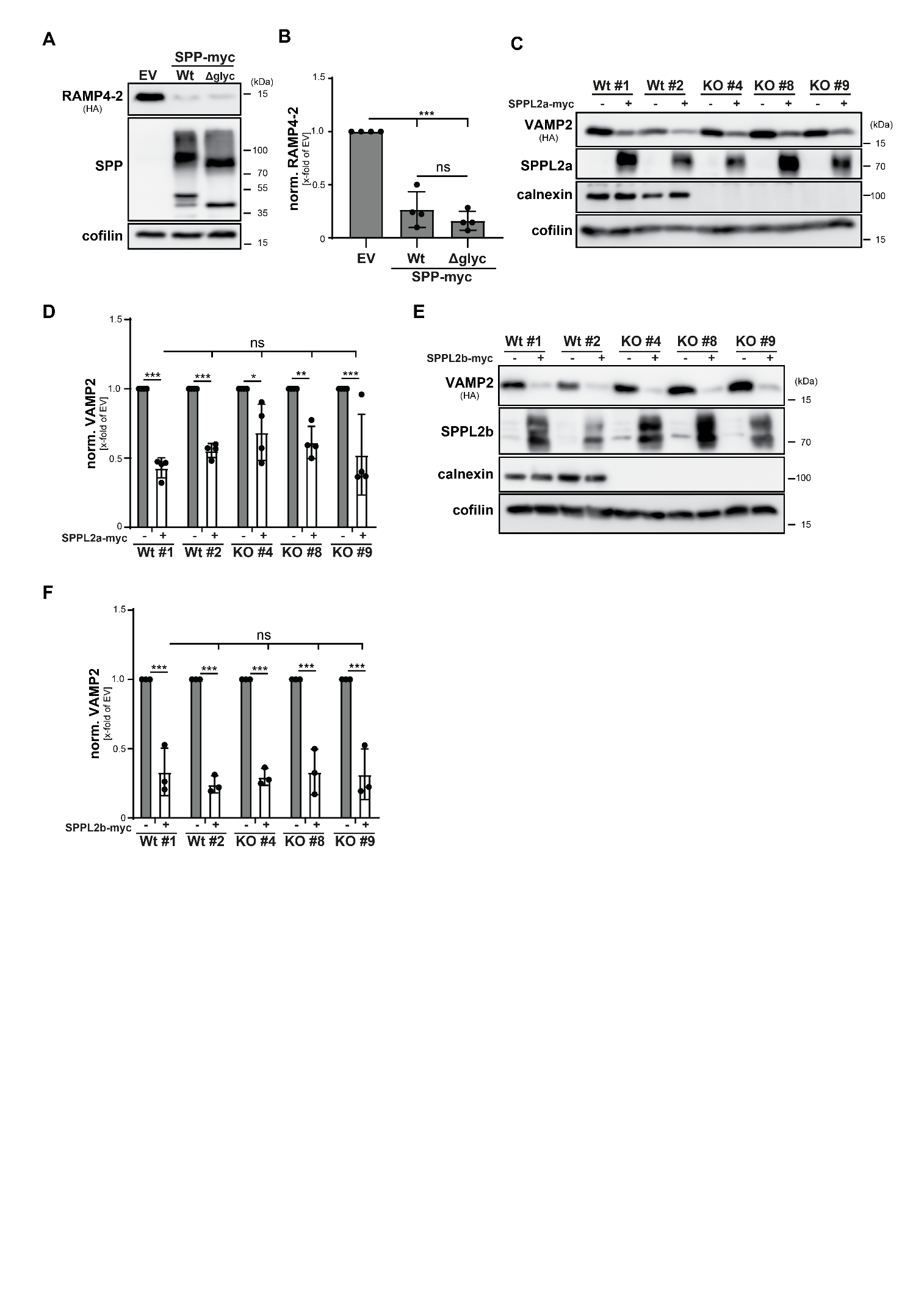


**Supplementary Figure 4. Calnexin-deficiency does not affect the proteolytic activity of SPPL2a and SPPL2b. A)** HEK293T cells were transiently transfected with HA-RAMP4-2 alone or together with either wild type (Wt) or glycosylation-deficient (N10/20A, Δglyc) SPP-myc. Processing of HA-RAMP4-2 by SPP was subsequently monitored by Western blotting. **B)** Quantification of A). N=4, n=4. One-Way ANOVA with Tukey’s post hoc test. **C)** Wild type (Wt) or calnexin-deficient (KO) HEK293T cells were transfected with the SPPL2a/b substrate HA-VAMP2 together with an empty vector (EV) or SPPL2a-myc. Processing of HA-VAMP2 by the intramembrane protease was subsequently evaluated by Immunoblotting. **D)** Quantification of C). N=4, n=4. Two-Way ANOVA with Tukey’s multiple comparisons test. **E)** The experiment described in A) was repeated with SPPL2b-myc instead of SPPL2a-myc. **F)** Quantification of E). N=3, n=3. Two-Way ANOVA with Tukey’s multiple comparisons test. ns, not significant; * p≤0.05; ** p≤0.01; *** p≤0.001.
